# Leukocytoclastic vasculitis as a cutaneous paraneoplastic syndrome in malignant mesothelioma

**DOI:** 10.1016/j.jdcr.2022.06.006

**Published:** 2022-06-19

**Authors:** Hyeok-Jin Kwon, Kyung-Deok Park, Jung-Ho Yoon

**Affiliations:** Department of Dermatology, College of Medicine, Dong-A University, Busan, Republic of Korea

**Keywords:** leukocytoclastic vasculitis, malignant mesothelioma, paraneoplastic syndrome, LCV, leukocytoclastic vasculitis

## Introduction

Leukocytoclastic vasculitis (LCV) is characterized by inflammation of superficial small vessels in the skin, manifesting as palpable purpura, erythema, wheals, or ulcerations.^1^ It can be induced by numerous factors including infections, drugs, autoimmunity, or malignancies.^1^ LCVs can occur as paraneoplastic syndromes related to various malignancies and take parallel paths depending on their clinical course.^2^, ^3^, ^4^ Although LCV is associated with several solid tumors, it has been more commonly noted in patients with hematologic cancers than in those with solid tumors.^3^^,^^4^ However, LCV has rarely been reported to occur associated with malignant mesothelioma compared with other solid tumors.^5^ We herein describe a case of LCV presenting as a paraneoplastic syndrome associated with malignant mesothelioma, which remarkably improved in parallel to the clinical course of the mesothelioma that effectively responded to chemotherapy.

## Case report

**Fig 1:**
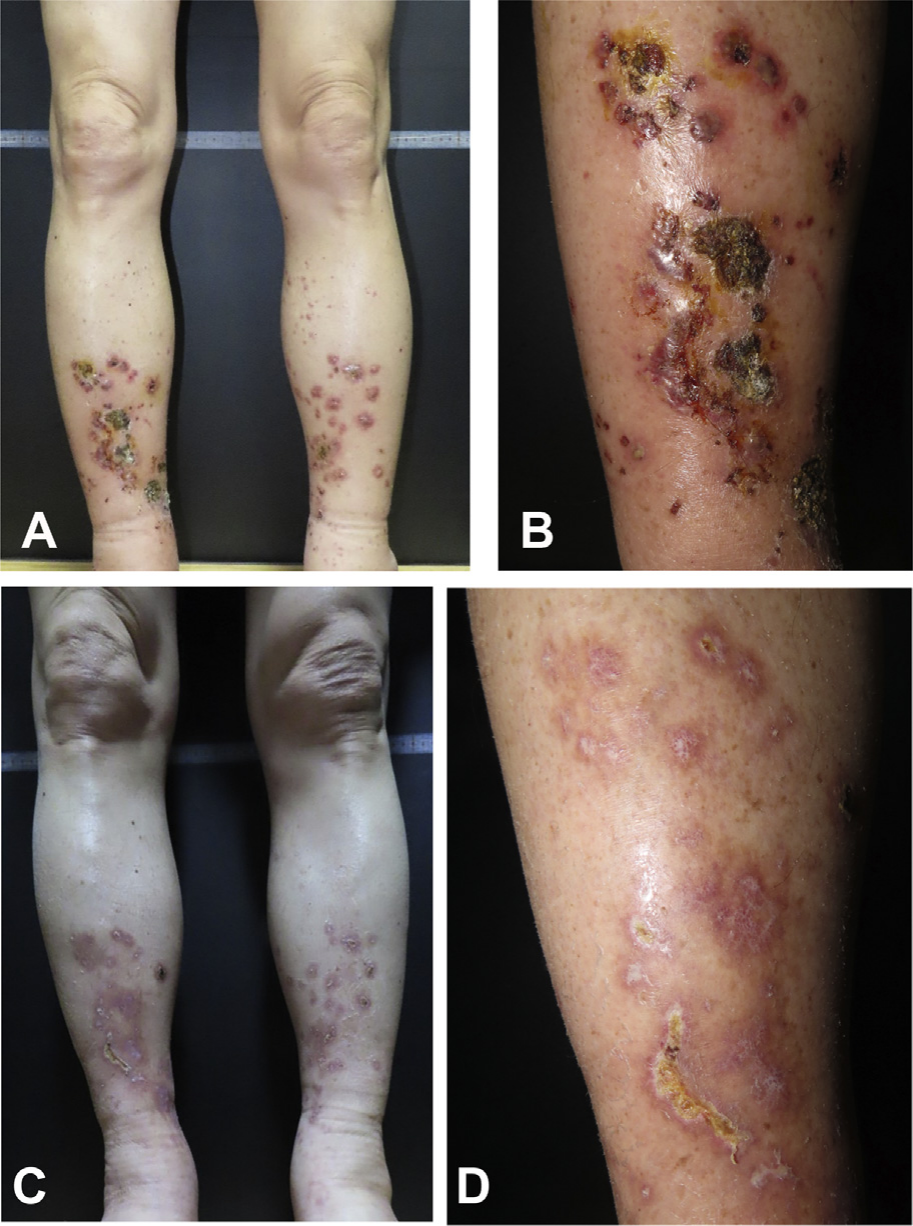
Cutaneous manifestations of the patient at the first visit and after chemotherapy. **A** and **B,** Multiple erythematous patches with ulcers accompanying exudates on the patient’s both legs. **C** and **D,** Cutaneous manifestations of the patient after a total of 10 chemotherapy cycles. During the chemotherapy period, ulcerative patches disappeared and erythematous patches partially improved.

**Fig 2:**
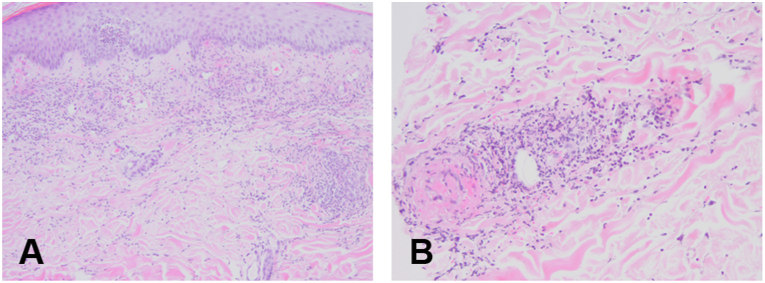
Histopathological findings of the initial cutaneous lesions on the leg. **A,** Hematoxylin and eosin staining showing superficial deposition of perivascular inflammatory cells. Intraepidermal abscess formation is observed. Original magnification, ×100. **B,** Hematoxylin and eosin staining showing typical findings of leukocytoclastic vasculitis presenting as dermal infiltrates predominantly composed of neutrophils with destructed vascular endothelial wall, extravasated erythrocytes, nuclear dust, and perivascular necrotic changes. Original magnification, ×200.

A 59-year-old Korean man visited our department due to multiple erythematous and ulcerative patches on both legs persisting for approximately 2 months (Fig 1, *A* and *B*). The patient was diagnosed with malignant mesothelioma without any metastasis 1 week before that visit. The patient reported long-term exposure to asbestos for 35 years at his workplace without an allergy or autoimmune disease history. Since the patient had not been previously treated for cutaneous lesions, we started treatment with prednisolone 10 mg twice a day, cetirizine 5 mg every day, and cefaclor 375 mg twice a day. However, no significant improvement was noted after 2 weeks of therapy. Tests including complete blood count, liver and kidney function, prothrombin time, activated partial thromboplastin time, complement, cryoglobulin, fibrinogen, C-reactive protein, blood culture, antithrombin III, D-dimer, rheumatoid factor, antinuclear antibody, anti–double-strand DNA antibody, and antineutrophil cytoplasmic antibody revealed no significant findings. A skin incisional biopsy revealed perivascular inflammatory cells in the dermis, comprising mainly neutrophils. At higher magnification, vascular wall destruction and necrotic changes were prominent (Fig 2, *A* and *B*).

**Fig 3:**
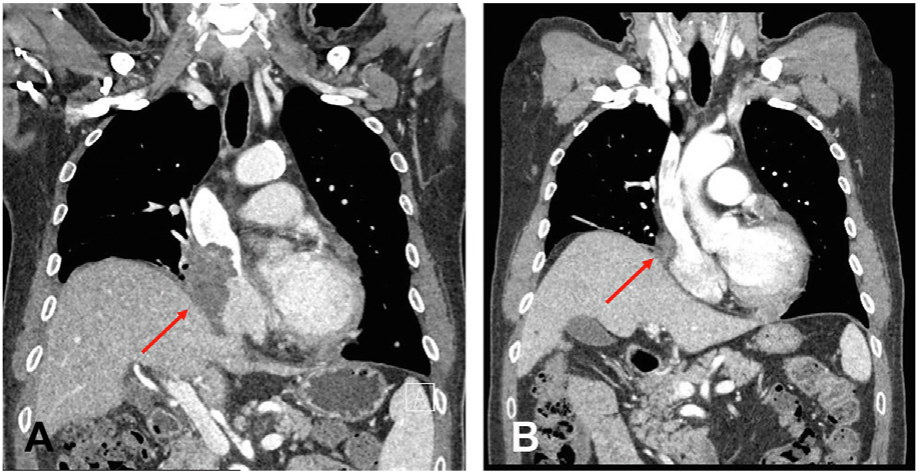
Computed tomography scan showing malignant mesothelioma. Computed tomography findings of a malignant pericardial mesothelioma **(A)** before chemotherapy (*arrow*) and **(B)** after 10 chemotherapy cycles (*arrow*).

Although the patient was diagnosed with LCV, the etiology remained obscure. Therefore, we transferred the patient to the oncologic department, suspecting that LCV could have originated from mesothelioma. The patient was then treated with pemetrexed (950 mg) and cisplatin (110 mg) per 3 weeks for a total of 10 cycles. During treatment, the tumor size significantly decreased (Fig 3, *A* and *B*). Moreover, the cutaneous lesions also partially improved in concordance with chemotherapy responses in mesothelioma, without the need for oral corticosteroids or antihistamines (Fig 1, *C* and *D*). No myelosuppression or other adverse effects were observed throughout the whole chemotherapy period. In the meantime, to confirm parallel clinical course between mesothelioma and cutaneous vasculitis, we additionally followed up the patient’s cutaneous lesions for 3 months after the last chemotherapy cycle. Consequently, no tumor progression or recurrent cutaneous vasculitis was noted over this additional 3-month period of observation. Based on these clinical courses, we hypothesized that LCV was derived from mesothelioma, manifesting as cutaneous paraneoplastic syndrome.

## Discussion

LCV formation can be induced by numerous factors including infection, drugs, and systemic diseases.^1^ Pathologically, patients with neutrophilic vascular damage with vascular wall degeneration commonly present with palpable purpura.^1^ LCV is also related to malignant processes usually discovered in various solid tumors as well as hematologic malignancies.^1^, ^2^, ^3^, ^4^ Despite this clinical evidence, establishing accurate correlation between cutaneous vasculitis and malignancies is still problematic for physicians. It has been reported that only 5% of all vasculitis cases were associated with malignancy.^3^ Therefore, several studies have recommended guidelines for vasculitis manifesting as the paraneoplastic process^2^^,^^3^^,^^6^: (1) temporal relationship, (2) consistency of clinical courses between malignancies and vasculitis, and (3) persistent vasculitis with poor response to conventional therapy, particularly in older patients. Accordingly, we suggested that the patient’s cutaneous manifestations were derived from the primary malignancy. This was based on the clinical history of vasculitis preceding the primary tumor diagnosis, failure of initial LCV therapy, and most importantly, both malignancies and cutaneous lesions have taken parallel clinical courses during the chemotherapy period. Additionally, we had not noticed any cutaneous vasculitis recurrence or tumor progression during an additional 3-month follow-up after the last chemotherapy cycle. Nevertheless, in our case, confirming consistency of clinical courses between malignancy and cutaneous vasculitis might be controversial due to the relatively short follow-up period. Furthermore, since “recurrent vasculitis presaging tumor recurrence or progression” could be stronger clinical evidence to prove parallel courses between malignancies and cutaneous vasculitis, our case might show potential limitations in this respect.^3^

Several studies have identified that LCV was associated with numerous solid tumors originating from the lung, digestive system, urinary tract, and even brain.^3^^,^^4^ However, to the best of our knowledge, only 1 case of LCV in a patient with malignant mesothelioma has been reported.^5^ Regarding mesothelioma, paraneoplastic manifestations present as glomerular diseases, sensorimotor polyneuropathies, oligoarthritis, or cerebellar degeneration.^7^, ^8^, ^9^ Among these manifestations, glomerular diseases are considered the most common paraneoplastic symptoms in patients with solid tumors.^7^^,^^10^ Despite the unclear pathophysiology, it has been suggested that tumor cell–related antigens and their antibodies may play significant roles in forming immune complexes.^8^^,^^10^

In agreement with this, we hypothesized that cutaneous LCV can be promoted by immune complexes originating from antigens triggered by primary tumor cells. Furthermore, it is well known that type III hypersensitivity and immune complex formation are the most significant mechanisms underlying LCV.^1^^,^^4^ We also considered that the patient’s cutaneous manifestations improved due to clearing immune complexes through tumor cell eradication, which corresponded with the clinical course of chemotherapy response against the primary malignancy. However, we could not prove this due to the relatively insufficient observation period that cannot confirm parallel clinical courses between malignancy and cutaneous manifestations.

In conclusion, despite possible limitations we described above, we report a case of LCV manifesting as paraneoplastic syndrome in a patient with malignant mesothelioma, which improved in concordance with chemotherapy response in the primary malignancy. This case shows clinical significance in that an insufficient response to conventional corticosteroid therapy against LCV could indicate undetected malignancies, particularly in older patients, which is also emphasized in other studies.^1^^,^^3^^,^^5^ Therefore, our case study not only describes LCV manifesting as paraneoplastic syndrome in a patient with mesothelioma but also indicates it as a meaningful clinical marker for detecting malignancies.

## Conflicts of interest

None disclosed.
